# Effect of probiotics on children with autism spectrum disorders: a meta-analysis

**DOI:** 10.1186/s13052-024-01692-z

**Published:** 2024-06-21

**Authors:** Ping Zeng, Cheng-zhi Zhang, Zhi-xing Fan, Chao-jun Yang, Wan-yin Cai, Yi-fan Huang, Zu-jin Xiang, Jing-yi Wu, Jing Zhang, Jian Yang

**Affiliations:** 1 Department of Cardiology, The First College of Clinical Medical Science, China Three Gorges University & Yichang Central People’s Hospital, Yichang, 443003 China; 2 Institute of Cardiovascular Diseases, Three Gorges University, Yichang, China; 3 Hubei Key Laboratory of Ischemic Cardiovascular Disease and HuBei Clinical Research Center for Ischemic Cardiovascular Disease, Yichang, China

**Keywords:** Probiotics, Autism spectrum disorder (ASD), Severity, Gastrointestinal, Meta-analysis

## Abstract

**Background:**

Researches have found that alteration of intestinal flora may be closely related to the development of autism spectrum disorder (ASD). However, whether probiotics supplementation has a protective effect on ASD remains controversial. This meta-analysis aimed to analyze the outcome of probiotics in the treatment of ASD children.

**Methods:**

The Pubmed, Cochrane Library, Web of Science and Embase were searched until Sep 2022. Randomized controlled trials (RCTs) relevant to the probiotics and placebo treatment on ASD children were screened. Quality assessment of the included RCTs was evaluated by the Cochrane collaboration’s tool. The primary outcomes were ASD assessment scales, including ABC (aberrant behavior checklist) and CBCL (child behavior checklist) for evaluating the behavior improvement, SRS (social responsiveness scale) for social assessment, DQ (developmental quotient) for physical and mental development and CGI-I (clinical global impression improvement) for overall improvement. The secondary outcome was total 6-GSI (gastrointestinal severity index).

**Results:**

In total, 6 RCTs from 6 studies with 302 children were included in the systemic review. Total 6-GSI (MD=-0.59, 95%CI [-1.02,-0.17], *P* < 0.05) decreased significantly after oral administration of probiotics. Whereas, there was no statistical difference in ABC, CBCL, SRS, DQ and CGI-I between probiotics and placebo groups in ASD children.

**Conclusion:**

Probiotics treatment could improve gastrointestinal symptoms, but there was no significant improvement in ASD.

**Supplementary Information:**

The online version contains supplementary material available at 10.1186/s13052-024-01692-z.

## Introduction

Autism spectrum disorder (ASD) is characterized by persistently impaired social communication and interaction [1]. The prevalence of ASD in the USA has increased significantly to 1 in 59 among 8-year-old children [2] and affects nearly 1% children attending elementary schools [3]. At present, the main treatment is direct communication interventions between therapist, child and parents [4]. However, the treatment effect of this method is not obvious, adding oral medication may be helpful, including nutritional supplement, anti-oxidant therapy, antipsychotics, deep brain stimulation and so on [5]. Studies have found that microbiota dysbiosis of the gastrointestinal system was believed to be implicated in the development of ASD [6, 7]. Interestingly, microbiome difference was found to be existed in ASD and healthy people [8]. It means that the gut microbiome composition and ASD are closely related. Clinical evidence has reported that the gut - brain axis was involved in the development and maintenance of ASD [9]. The mechanism may be as follows, short-chain fatty acids maybe binding to or activating free fatty acid receptors expressed on the vagus nerve, thus affect the olfactory system and immune system. On the other hand, probiotics can influence the central nervous system through regulating the secretion of oxytocin in pituitary gland and cortisol in adrenal gland [10]. In recent years, more and more studies on gut-brain disorder-related therapies, such as probiotics supplement, have confirmed that it may be effective for ASD children. A meta-analysis showed that probiotics and prebiotics did not significantly improve the severity of ASD patients [11]. However, the inclusion of three RCTs limited the persuasiveness of the analysis. Moreover, there exist several studies which showed different results recently, thus we added several new RCTs on top of that to make this meta-analysis.

## Methods

### Literature search strategy

We followed the method proposed by the Preferred Reporting Items for Systematic Reviewsand Meta-Analyses flow diagram (PRISMA) guidelines. Pubmed, Cochrane Library, Web of Science and Embase were used in the retrieval process. Probiotics, autism spectrum disorder were searched as the key words by the combination of medical subject headings (MeSH) and entry term in English, and all literatures were searched in the database until Sep 2022. Search strategy for Pubmed and other databases were described in the supplement document. This meta-analysis carried out to the standards established by the PRISMA recommendation (Preferred Reporting Items for Systematic Reviews and Meta-Analysis).

### Literature inclusion and exclusion criteria

Literature inclusion criteria: [1] patients diagnosed with ASD; [2] patients who were treated with probiotics and placebo; [3] RCT studies. Exclusion criteria: [1] reviews, comments, case report and animal experiment; [2] literature that lack of clinical trial data; [3] literature with duplicate data; [4] not RCT articles. This systematic review was performed by two authors who independently judged whether the retrieved literature could be included in the study, and the third author need to make an independent judgment whether to include it or not in case of disagreement.

### Outcome measures

Primary outcome: the severity of ASD was evaluated by several rating scales, including ABC (aberrant behavior checklist) [12], CGI-I (clinical global imprssion- improvement) [13], SRS (social responsiveness scale) [14] and CBCL (child behavior checklist) [15].The higher the scores, the more serious the condition of autism. DQ (developmental quotient) [16] indicates physical and mental development. Secondary outcome: the severity of gastrointestinal symptoms was evaluated by total 6-GSI (gastrointestinal severity index) [17]. The higher the level of 6-GSI, the more serious the gastrointestinal condition.

#### Literature quality evaluation criteria

**Fig. 1:**
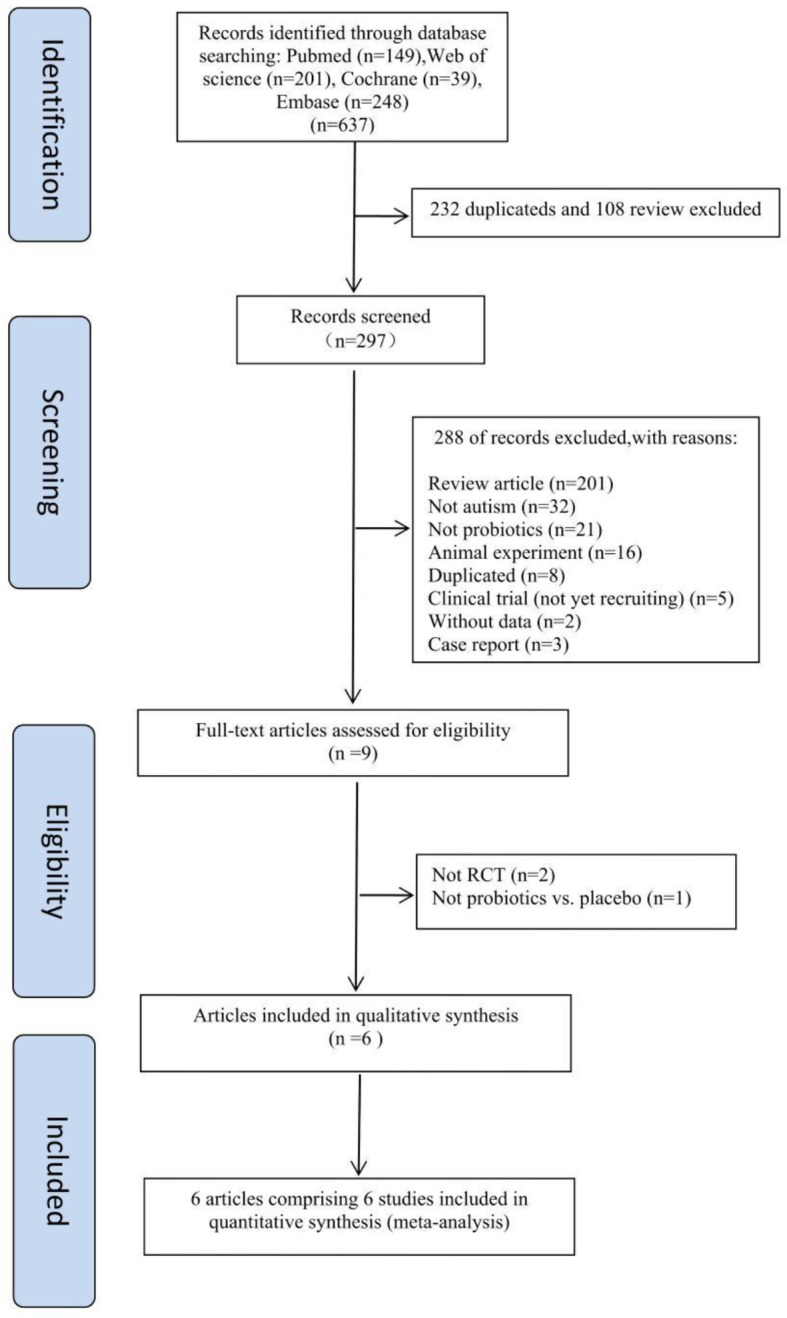
Flowchart of study selection process in the Meta-analyzes

The quality assessment was evaluated by the Cochrane collaboration’s tool. The result was provided by the Revman 5.3, which was used to evaluate the quality of the RCTs. (Fig. 1).

### Data extraction

The extracted data included the essential information of the study included authors, year of publication, age, gender composition, body mass index (BMI), weight, height, types of probiotics and ASD related index. Data that could not be extracted directly could be obtained by data transformation.

### Statistical methods

Statistical analysis of the data was performed using Revman 5.3. Forest plots were used to present the combined effect quantity of the odds ratio (OR) and 95% confidence interval (CI). Funnel map was used to evaluate publication bias. The heterogeneity was evaluated using the I-square (I^2^) statistic, I^2^ < 50% indicated no significant heterogeneity among studies, so the effect indicators were combined using the fixed-effect model. I^2^ > 50% indicated significant heterogeneity, the randomized effect model was then adopted. The software of R was used for sensitivity analysis, and *P value* < 0.05 was considered a statistically significant difference.

## Results

### Literature search results

**Fig. 2:**
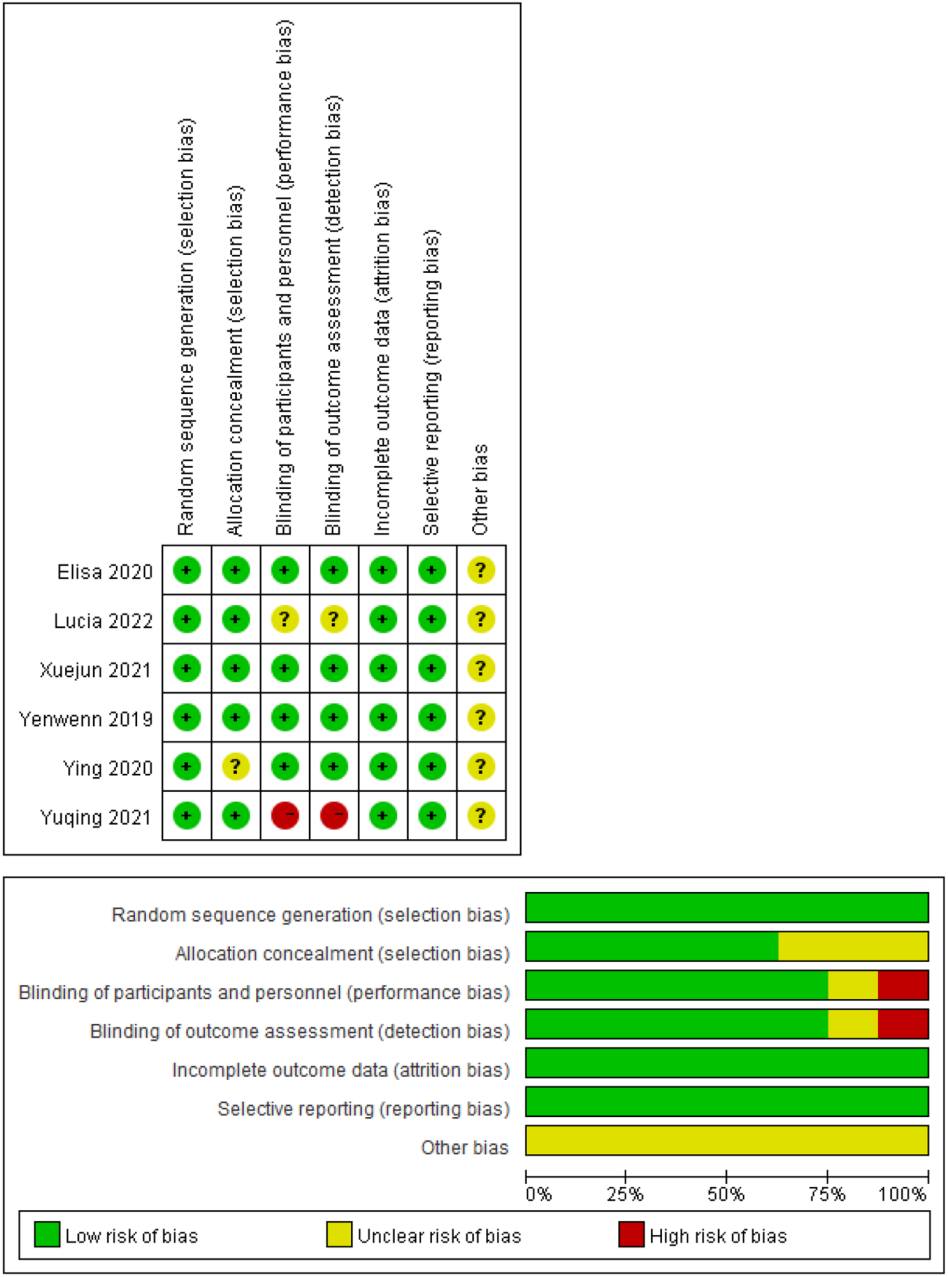
Publication bias of this meta-analysis

Six hundred and thirty-seven articles were obtained by searching with the proposed input. 232 repetitive articles and 108 review type articles were then excluded by reading the titles and abstracts. 297 articles entered into the next step of retrieval, 288 articles were initially excluded (201 were review articles; 32 were not about autism; 21 were not about probiotics; 16 were animal experiments; 8 were duplicates; 5 were clinical trials that were not yet recruiting; 2 without data; 3 were case reports; 2 were not RCTs; 1 was not probiotic vs. placebo) according to the inclusion and exclusion criteria. (Fig. 2).

### Basic characteristics of the included literature

6 RCTs were included after carefully reading the full texts [18–24]. The basic information of the included studies was shown in Table 1. Total sample sizes of 302 patients were included. The studies were performed in Italy, USA, Taiwan and other cities in China. The mean age of the participants in each studies ranged from 3 to 14 years old, the percentage of boys ranged from 64 to 92%, and the mean BMI (body mass index) varied from 14 to 22 kg/m2 (Table 1). Baseline indexes comparison were presented in forest maps and all p value > 0.05 (see Supplementary Materials).

**Table 1:** Characteristics of included studies

Author	Year	Country	Treatment duration	Groups	Treatment	Number	Age (years)	Boys (*n*,%)	BMI	Weight	Hight	ASD related index
Elisa	2020	Italy	6 months	Probitics	Contain eight probiotic strains: Streptococcus thermophilus, Bifidobacterium breve, Bifidobacterium longum, Bifidobacterium infantis, Lactobacillus acidophilus, Lactobacillus plantarum, Lactobacillus para-casei, Lactobacillus delbrueckii subsp	42	4.16 ± 1.17	34(80.9)	15.93 ± 1.73			DQ: 64.6 ± 16.4 vs. 60.5 ± 19.1; CBCL: 61.5 ± 9.9 vs. 62.9 ± 10.8; 6-GSI: 2.3 ± 2.2 vs. 1.8 ± 1.6
				Placebo	Placebo	43	4.13 ± 1.0	37(86)	15.98 ± 1.62			
Ying	2020	China	30 days	Probitics	Bifidobacterium infantis Bi-26, Lactobacillus rhamnosus HN001, Bifidobacteriumlactis BL-04, Lactobacillus paracasei LPC-37	16			18.39 ± 1.22	20.73 ± 1.56	1.07 ± 0.18	6-GSI: 4.88 ± 0.43 vs. 3.8 ± 0.46; ATEC: 85.06 ± 5.72 vs. 78.2 ± 5.49
				Placebo	Placebo	8			17.45 ± 5.55	20.75 ± 5.87	1.11 ± 0.22	
Yenwenn	2019	Taiwan China	4 weeks	Probitics	PS128	36	10.11 ± 2.34			18.66 ± 6.78	1.44 ± 0.16	CBCL: 49.63 ± 25.4 vs. 50.6 ± 25.91; SRS: 138.87 ± 24.19 vs. 135.88 ± 26.04
				Placebo	Placebo	35	9.91 ± 2.33			17.83 ± 7.56	1.41 ± 0.15	
Lucia	2022	Italy	6 months	Probitics	Streptococcus thermophilus, Bifidobacterium breve, Bifidobacterium longum, Bifidobacterium infantis, Lactobacillus acidophilus, Lactobacillus plantarum, Lactobacillus para-casei, Lactobacillus delbrueckiisubsp.	26	4.4 ± 1.29	20(76.9)				DQ: 66.1 ± 17.8 vs. 66.9 ± 21.4; 6-GSI: 2.1 ± 2.2 vs. 1.7 ± 1.6
				Placebo	placebo	20	3.78 ± 0.86	15(75)				
Yuqing	2021	China	3 months	Probitics	Bifidobacterium triple viable powder	21	4.6 ± 1.7	16(76)				ATEC: 84 ± 27 vs. 82 ± 27
					placebo	20	4.5 ± 1.8	15(75)				
Xuejun	2021	USA	28 weeks	Probitics	PS128	18	9.85 ± 4.91	15(83.3)				ABC: 272 ± 30.2 vs278 ± 34.8(T score); SRS: 82.3 ± 11.5 vs. 83.0 ± 12.1
				Placebo	placebo	17	10.7 ± 4.76	11(64.7)				

BMI, body mass index; PS 128, Lactobacillus plantarum PS1; ABC, aberrant behavior checklist; CBCL, child behavior checklist; SRS, social responsiveness scale; DQ, developmental quotient; CGI-I, clinical global impression improvement; total 6-GSI, gastrointestinal severity index

### Quality of evidence and risk of bias across studies

The quality of RCTs were presented in Fig. 2, the red button represents “high risk”, yellow button represents “unknown risk” and green button represents “low risk” (Fig. 2). Most of the studies showed high quality with strict randomization, double-blind and assignment concealment. The yellow percentage was high because the source of other bias was not described in the articles. Yuqing 2021 et al. didn’t describe whether it was double-blind or not, thus resulted in more red parts and brought heterogeneity.

## Outcomes

### ASD assessment scales

**Fig. 3:**
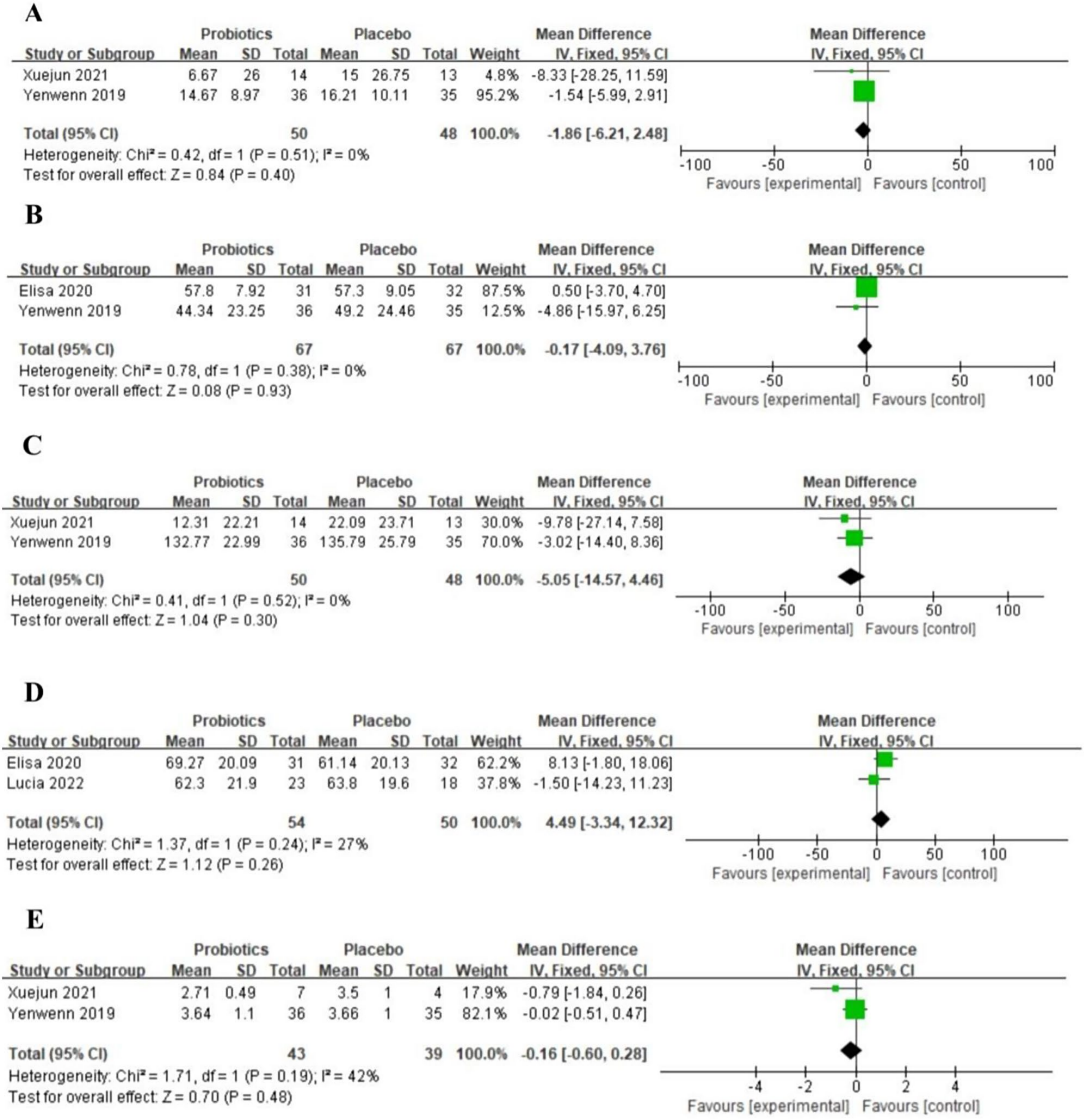
Forest plot of ABC(**A**), CBCL(**B**), SRS(**C**), DQ(**D**) and CGI-I (**E**) in ASD children treated with probiotics vs. placebo. ABC, aberrant behavior checklist; CBCL, child behavior checklist; SRS, social responsiveness scale; DQ, developmental quotient; CGI-I, clinical global impression improvement

Scales of ABC (MD=-1.86, 95%CI [-6.21, 2.48], *P* = 0.4) (Fig. 3A) and CBCL (MD=-0.17, 95%CI [-4.09, 3.76], *P* = 0.93) (Fig. 3B) showed no statistical significance in behavior improvement. The scale of SRS (MD=-5.05, 95%CI [-14.57, 4.46], *P* = 0.3) (Fig. 3C) suggested that probiotics supplementation cannot effectively improve the social ability of children with ASD. And also, there was no significant increase of DQ (MD = 4.49, 95%CI [-3.34, 12.32], *P* = 0.26) (Fig. 3D). Probiotics therapy could not significantly contribute to overall improvement in CGI-I (MD=-0.16, 95%CI [-0.60, 0.28], *P* = 0.48) (Fig. 3E).

### The severity of gastrointestinal symptoms

**Fig. 4:**
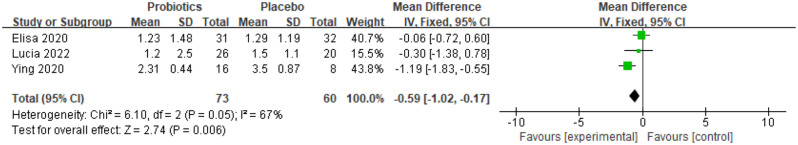
Forest plot of total 6-GSI in ASD children treated with compound probiotics vs. placebo

Probiotics treatment was associated with lower severity of gastrointestinal symptoms, the total 6-GSI in probiotics group was lower than that of the placebo group (MD=-0.59, 95%CI [-1.02,-0.17], *P* = 0.006) (Fig. 4).

## Discussion

We conducted a systemic meta-analysis of RCTs about probiotics treatment among children with ASD. After conducting the meta-analysis, we found that probiotics treatment showed beneficial effect in alleviating gastrointestinal symptoms, but cannot improve ASD severity. In this study, ABC, CBCL, SRS, DQ, and CGI-I were scales for assessing the severity of ASD symptoms. We found that all of these scores showed no significant change in probiotics and placebo groups. SONG and his colleagues [11] discovered that neither probiotics nor prebiotics exerted a significant improve on the severity of symptoms, gastrointestinal issues, or comorbid psychopathology in individuals with ASD. However, our study findings indicate that although probiotics did not improve the severity of ASD, they did significantly ameliorate gastrointestinal symptoms.

Some studies have proved that probiotics does help improve ASD symptoms. Hsaio and his colleagues [25] showed that ASD symptoms were triggered by compositional and structural shifts of microbes and associated metabolites. Probiotics may provide therapeutic strategies for neurodevelopmental disorders by lowering the level of 4-ethylphenylsulfate (4EPS), which has been presented as a human autism biomarker [26]. Probiotics have an advantage in modulating brain development and behavior through the gut microbiota-brain axis, which involved a variety of mechanisms including immune, neural, and metabolic pathways [27]. Moreover, gut microbes breakdown indigestible carbohydrate fibers and convert them into short-chain fatty acids (SCFAs), which could act locally to support intestinal epithelial function. Thus induce hormone and neuropeptide production, such as glucagon-like peptide 1 (GLP-1), peptide YY (PYY), and histone deacetylase inhibitors from intestinal enteroendocrine cells and influence the processes of learning and memory ultimately [28, 29].

However, our study found that probiotics supplementation did not improve the severity of ASD symptoms, and we analyzed the possible reasons as follows. First of all, the pathophysiological mechanism of ASD was very complex, and multiple factors were involved. It was far from enough to rely only on probiotics supplementary based on direct communication therapy. Secondly, probiotics included compound probiotics and single probiotics. In our study, The children in ABC, SRS and CGI-I scales were given single probiotics (PS128), while the two RCTs included in DQ were given compound probiotics, and objects included in CBCL were supplemented by both single and compound probiotics. Different types of probiotics may affect the outcome, and this may have contributed to the negative results. Thirdly, the dosage of probiotics and the manufacturer were not specified. If the children have diarrhea, constipation or other conditions, the absorption of probiotics may be different.

Nevertheless, we believe that the meta-analysis is still of great significance. There was data that children with ASD were 4 times as likely to experience gastrointestinal symptoms as those without. In ASD children, there’s a notable decline in the ratio of Bacteroidetes to Firmicutes, coupled with a significant elevation in lactobacilli levels. Intriguingly, supplementation with probiotics has demonstrated improvements in the behaviors of these children, notably reducing destructive tendencies and anxiety, while enhancing social aptitude and cognitive function [30]. Thus, probiotics and ASD may be closely related. In our study, we found significant improvement in gastrointestinal function with a lowered total 6-GSI in the probiotics group. On the one hand, probiotics contain microorganisms, most of which were similar to the beneficial bacteria that occur naturally in the human gut. Studies have proved that probiotics were effective for acute infectious diarrhea, antibiotic-associated diarrhea and clostridium difficile- associated diarrhea [20]. On the other hand, Gut microbiome was able to communicate with brain activities through microbiota-derived signaling molecules, immune mediators, gut hormones as well as vagal and spinal afferent neurons [31]. We found that probiotics did improve several ASD assessment scales, but the differences were not statistically significant. Ning Sun et al. have proved that compound probiotics could improve body growth performance by enhancing intestinal development [32]. While, another study stated that probiotics could be effective in reducing body mass index and hip circumference, which may be harmful to children’s growth and development [33]. The role of probiotics in children with ASD is still controversial, which is really worth studying.

Although this meta-analysis included a relatively comprehensive literature search and high quality RCTs, there were still some limitations. First of all, the strains in the probiotics group were inconsistent in each included RCTs, which may lead to heterogeneity. Secondly, the observation time included in the study was inconsistent. Different treatment duration of probiotics may affect the outcomes of the study and resulting in heterogeneity, as we observed in CGI-I and 6-GSI. Thirdly, both complex and single probiotics were used in the studies we included, some probiotics consist of a variety of beneficial bacteria, while others have only a single strain, such as PS128. Fourthly, all RCTs were published in different countries, the researches were scattered in Asia, Europe and the United States, race and ethnicity maybe important influencing factors of autism [34]. Higher quality and larger sample size clinical studies are needed for further study.

## Conclusion

Our systematic review and meta-analysis found a relationship between probiotics treatment and ASD children, probiotics supplementation could improve gastrointestinal symptoms. But there was no statistically significant difference in behavioral, social, physical and mental development and overall improvement in ASD children.

### Electronic supplementary material

Below is the link to the electronic supplementary material.

Supplementary Material 1

Supplementary Material 2

## Data Availability

All data generated or analyzed during this study are included in this published Article.

## Abbreviations

ASD: Autism spectrum disorder
RCTs: Randomized controlled trials
ABC: Aberrant behavior checklist
CBCL: Child behavior checklist
SRS: Social responsiveness scale
DQ: Developmental quotient
CGI: I-Clinical global impression improvement
6: GSI-Gastrointestinal severity index
BMI: Body mass index
4EPS: 4-ethylphenylsulfate
SCFAs: Short-chain fatty acids
GLP: 1-Glucagon-like peptide 1
PYY: Peptide YY
