# Supplementary figures and images for: Effect of probiotics on children with autism spectrum disorders: a meta-analysis
Source: Ital J Pediatr. 2024 Jun 21;50:120. doi: 10.1186/s13052-024-01692-z (PMC11191217; doi:10.1186/s13052-024-01692-z)

Scores of ASD children in the two groups before intervention (all *p* <0.05)


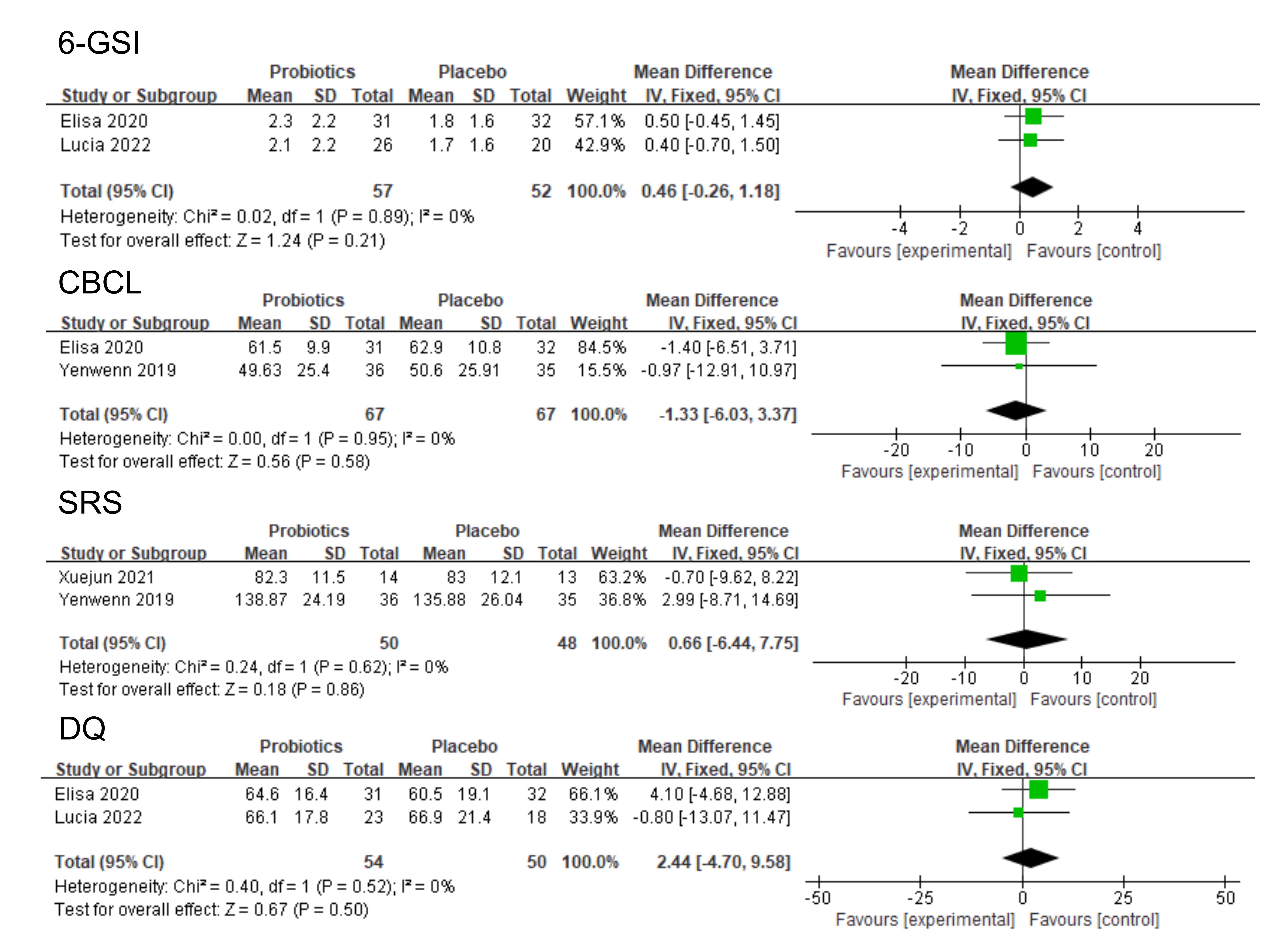

Supplement: Supplementary file 1 — Supplementary Material 1 [file 13052_2024_1692_MOESM1_ESM.doc]

Pubmed (n=149)


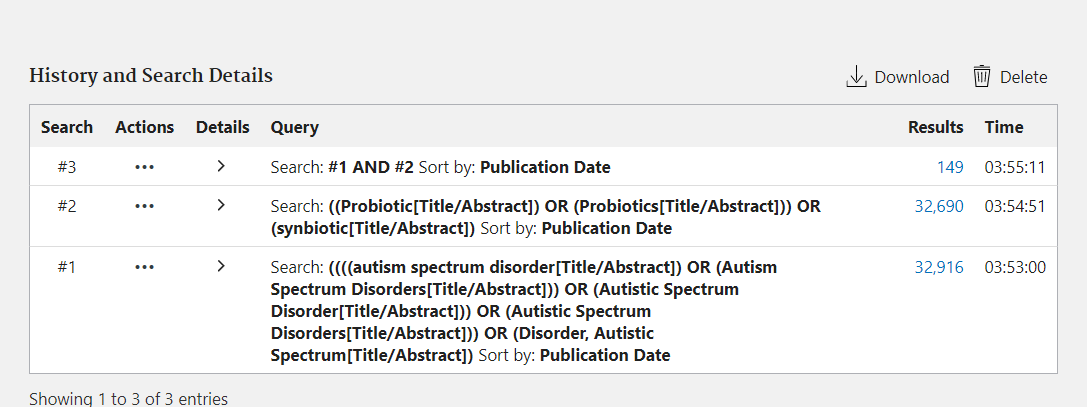


Web of science (n=201)


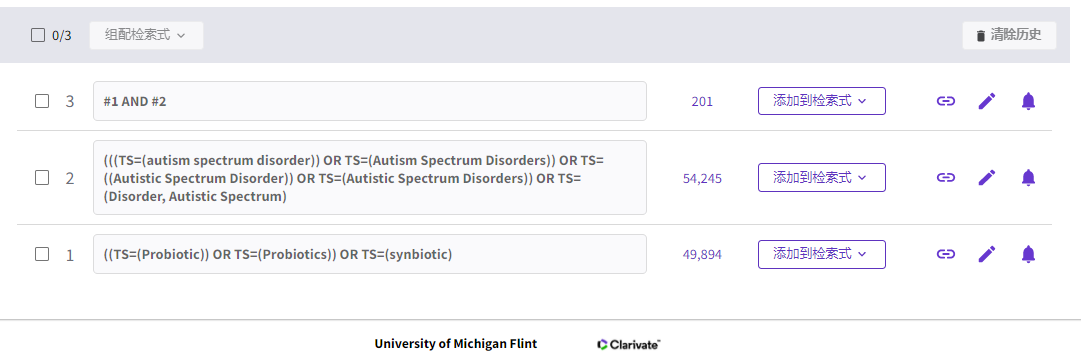


Cochrane (n=39)


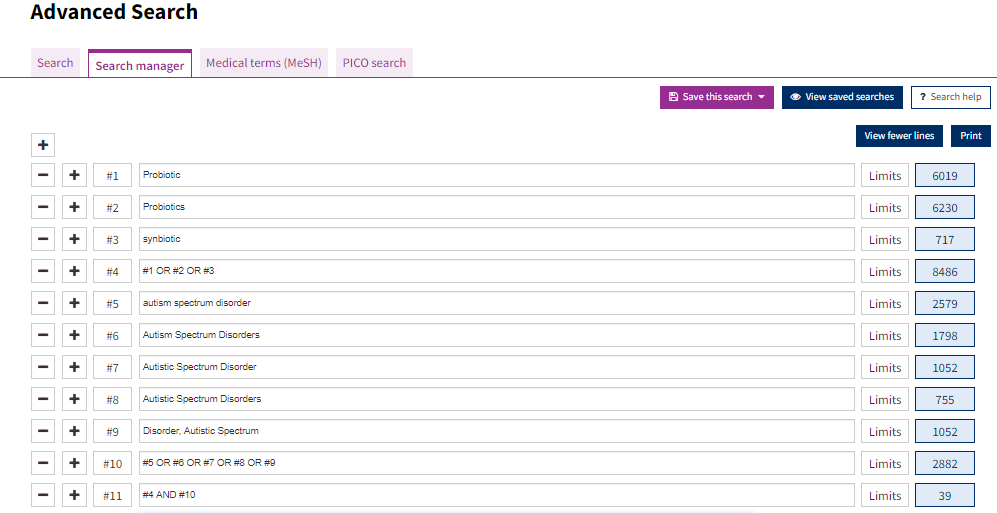


Embase (n=248)


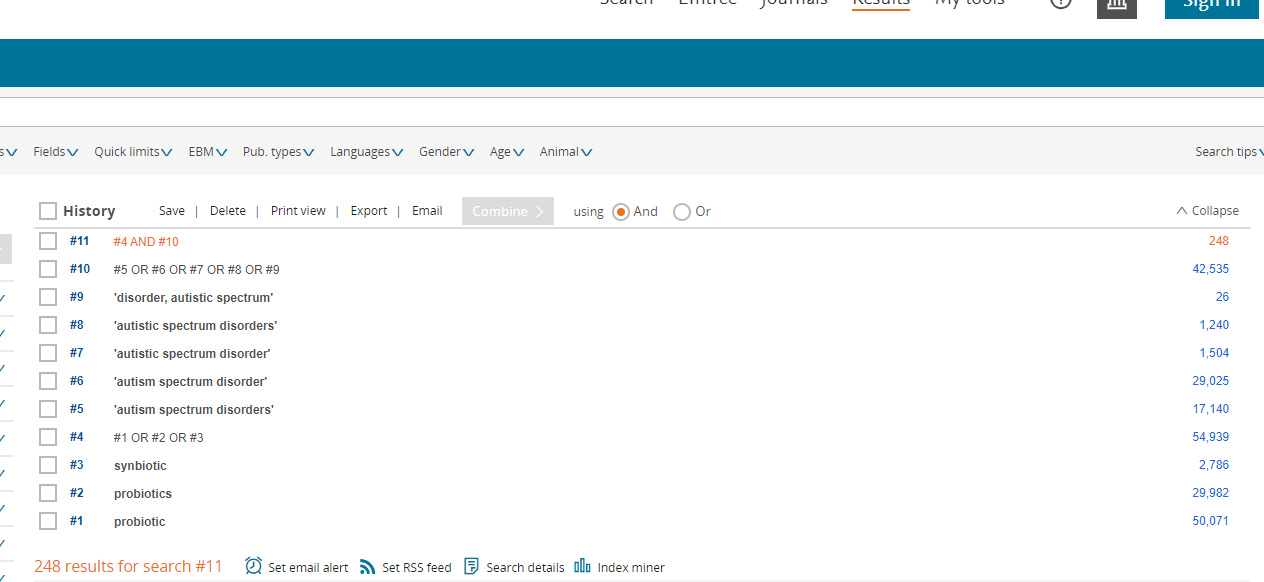

Supplement: Supplementary file 2 — Supplementary Material 2 [file 13052_2024_1692_MOESM2_ESM.doc]
